# Effectiveness of a tailored communication intervention to improve physical activity in hypertensive patients: a twelve-month randomized controlled trial

**DOI:** 10.1186/s12872-024-03786-2

**Published:** 2024-03-05

**Authors:** Roberta Adorni, Francesco Zanatta, Erika Rosa Cappelletti, Andrea Greco, Patrizia Steca, Marco D’Addario

**Affiliations:** 1 Department of Psychology, University of Milano-Bicocca, Milan, Italy; 2 Health Promotion Division, Agenzia Tutela Salute Milano, Milan, Italy; 3 Department of Human and Social Sciences, University of Bergamo, Bergamo, 24129 Italy

**Keywords:** Physical activity, Lifestyle, Health Communication, Hypertension, Cardiovascular diseases, Randomized Clinical Trial

## Abstract

**Background:**

Cardiovascular diseases (CVDs) are a leading cause of morbidity and mortality globally. Arterial hypertension is one of the main cardiovascular risk factors. Despite this, individuals with hypertension often fail to follow medical advice to counteract cardiovascular risks. A physically active lifestyle is one of the most challenging behaviors to adopt. This study aimed to preliminarily investigate the effectiveness of a tailored print message intervention to increase physical activity in patients with arterial hypertension.

**Methods:**

A sample of 188 patients with hypertension (mean age = 63 years; SD = 10.9; 50% men) participated in a theory-based tailored health communication intervention. Participants were randomly assigned to three parallel groups: (1) the tailored group, which received tailored health brochures; (2) the non-tailored group, which received non-tailored health brochures; or (3) the usual care group, which received no informative print materials. The longitudinal physical activity trajectories (baseline, 6- and 12-month follow-ups) were examined using repeated measures ANOVA and growth curve models.

**Results:**

At the baseline, 38.8% of patients achieved the target physical activity. This percentage rose to 43.0% at 6-month follow-up and 46.0% at 12-month follow-up. The descriptive statistics divided in function of the experimental group suggested differences in the longitudinal trend of the mean physical activity depending on the experimental group. However, statistical significance using repeated measures ANOVA did not support this observation. The analysis of the growth curves suggested that the tailored group showed a progressive increase in physical activity over twelve months (the model that best described the longitudinal trajectory was a linear growth model). The non-tailored group showed an increase in physical activity six months after the beginning of the intervention, followed by a decrease (free time score model). The usual care group did not change over time (no-growth model).

**Conclusions:**

Findings suggest the effectiveness of the tailored intervention proposed. However, further investigations and empirical confirmations are required.

**Trial registration:**

ISRCTN13415993 (10.1186/ISRCTN13415993). Registration date: 08/04/2019.

## Introduction

Cardiovascular diseases (CVDs) are one of the leading causes of death worldwide [1]. In particular, arterial hypertension is one of the primary and most widespread cardiovascular risk factors, and it is still rising [2]. International guidelines have been drawn up for diagnosing and treating arterial hypertension. All of them agree in considering the change in lifestyle an essential element for preventing or managing the disease [3]. For example, World Health Organization (WHO) considers sedentary behavior as one of the leading causes of global mortality, especially in individuals presenting concomitant metabolic risk factors or unhealth conditions, and that higher attention on the strategies to foster physical activity is therefore required [4]. Contextually, this goal is considered reachable when adequate knowledge of the process of behavioral change that leads sedentary individuals to adopt physical activity and maintain it over time is considered. However, individuals with hypertension often fail to change their unhealthy habits [2, 5].

According to prior studies, this may be linked to the fact that arterial hypertension has few or no symptoms and a low impact on individuals’ everyday life. Therefore, individuals with hypertension often underestimate their risk and “normalize” their condition, leading them to be little prone to change their lifestyles [5].

In this context, education in adopting a healthy lifestyle is a priority. Given the low propensity of patients with hypertension to adopt a healthy lifestyle, it is essential to identify health-related communication strategies that can promote improved behavior.

The greatest challenge when it comes to health education is the development and delivery of messages that are not only informative but also relevant, interesting, informative, and persuasive. Tailored print communication based on recipients’ needs, preferences, and personal characteristics is an example of a communication approach that has attempted to address this challenge and has been applied in health promotion [6, 7].

Tailored communication can be defined as a combination of communication strategies to stimulate behavioral change in a specific individual based on assessing that individual’s unique characteristics [7]. Accordingly, tailored messages can facilitate behavior changes by providing personally relevant information and feedback [8, 9]. Contextually to health promotion field, the construction of tailoring protocols is usually based on different behavioral change models.

One of the most influential theoretical models in designing tailored communication interventions is the Health Action Process Approach (HAPA) [10]. The HAPA defines the implementation and maintenance of the health behavior as the outcome of a staged process. Each stage sees the intervention of specific socio-cognitive variables, which can facilitate or hinder, the entire process. In particular, during the first “motivational” phase, three antecedents can act in the development of the intention to change: (a) the risk perception related to the current unhealthy behavior; (b) the outcome expectancies associated with the newly adopted behavior; and the level of confidence in one’s ability to effect the change (i.e., action self-efficacy). Once the behavioral intention has been matured, individuals enter the subsequent “volitional” phase, where additional factors come into play: (a) planning, which allows to mentally simulate successful scenarios on when, where, and how to implement the change (i.e., action planning), and to generate strategies to overcome any obstacles to action (i.e., coping planning); (b) the level of confidence in one’s ability to face any obstacles and maintain the new behavior (i.e., maintenance self-efficacy); (c) and the level of confidence in being able to face any setbacks and overcome failures, returning to enact the desired behavior (i.e., recovery self-efficacy) [10].

The implementation of the HAPA model has been demonstrated useful and valid for various health behaviors [11]. Particularly, physical activity change was investigated as one of the major topics within the HAPA framework. Plenty of studies have been conducted on both healthy [12] and clinical populations [13], specifically with individuals after a cardiac event [14].

Despite the solid theoretical basis, increasing physical activity still remains the most difficult behavioral change to implement [15]. Prior research showed, however, encouraging results about the effectiveness of health communication intervention through the implementation of tailored messages in helping individuals to increase their physical activity. For instance, two meta-analyses [16, 17] reported small but significant effects of tailored print message interventions in promoting physical activity among adult populations, concluding that tailored contents are more effective that non-tailored ones. More recently, other studies have highlighted the usefulness of these type of interventions delivered via mobile technology (for example, see the systematic review by Davis and collaborators) [18].

Despite the encouraging results, little is known about the long-term effectiveness of tailored messages to improve physical activity in patients with hypertension, especially through the adoption of a theory-based approach. A large majority of studies on tailored intervention mainly adopted a cross-sectional study design or explored the short-term effects of tailored messages, while few studies investigated long-term effects but mostly up to 6 months after the intervention [16–18].

In order to fill this gap in the literature, a theory-based, randomized, controlled, tailored communication intervention protocol aimed at improving the lifestyle of patients affected by this pathology was designed and implemented [19]. Tailoring was designed on the HAPA [10], and the intervention was delivered through printed materials. The choice to implement printed materials was preferred and driven by prior research on Italian patients with hypertension, suggesting that this segment of the population considers more relevant print health material given by physicians than the internet [20]. Moreover, printed materials offer the benefit of good scalability and accessibility regardless of educational level, especially considering that patients with hypertension are usually of advanced age and, in some cases, may be little familiar with mobile technology [21].

This study aimed to evaluate the effectiveness of this intervention in supporting patients with hypertension in increasing their physical activity over 12 months.

Based on prior research [16, 17], this study tested the hypothesis that patients receiving the tailored printed messages significantly improve physical activity over time. Particularly, a significant effect of the intervention is hypothesized with the patients receiving the tailored printed messages that show significant wider improvements in their physical activity over 12 months compared to patients receiving non-tailored printed messages or no printed materials.

## Methods

### Participants and procedure

**Fig. 1:**
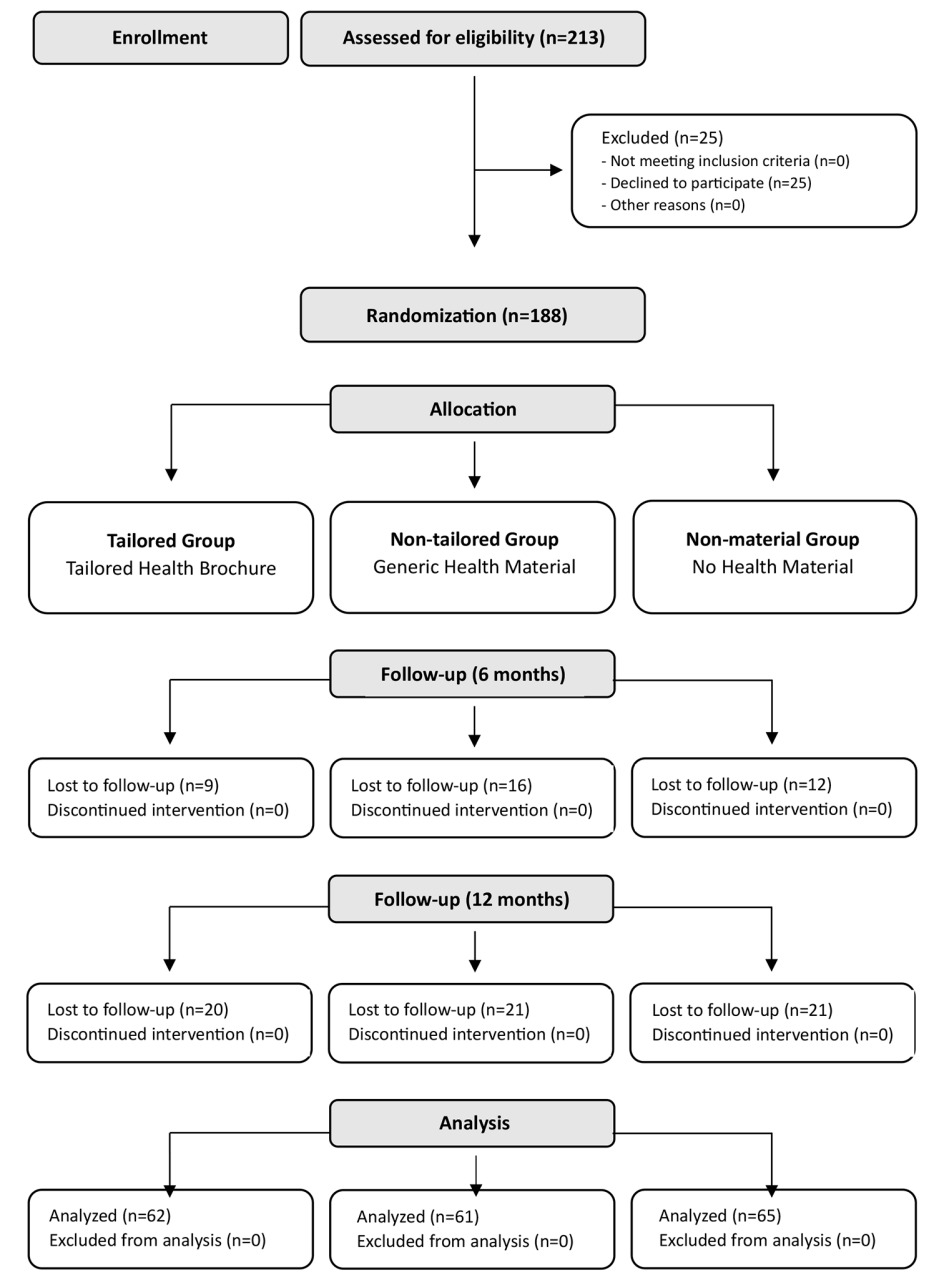
Flow chart of the study

The study has longitudinal three-arm, parallel group, randomized study design and included three assessments: baseline (t0), six (t1), and twelve-month follow-ups (t2) (Fig. 1).

It was carried out at two medical centers treating arterial hypertension in Milan. Participants were considered eligible according to the following criteria: age over 18 years, a diagnosis of primary arterial hypertension (SBP ≥ 140 mmHg or DBP ≥ 90 mmHg) under regular pharmacological treatment, sufficient knowledge of the Italian language, no cognitive deficit, no comorbidity with other significant pathologies (e.g., cancer or cardiovascular disease). In particular, patients were excluded if having a particular pharmacological treatment that prevented them from voluntarily changing their lifestyle without an indication from the treating physician.

A physician consecutively informed eligible patients about the study during the specialist visits. Thereafter, interested patients signed a written consent to participate. The physician collected clinical information about risk factors (dyslipidemia, smoking, and diabetes).

At baseline, the patients were asked to fill out a battery of questionnaires under the supervision of a psychologist. The battery and the intervention protocol are fully described elsewhere [19].

Before participants recruitment, an independent researcher generated and sealed computerized randomization sequences in stacks of three and group allocation was determined by drawing a sealed envelope per patient. Following baseline data collection, each participant was then assigned to one of the three groups. The “Non-Tailored Group” (NT) received informative brochures about hypertension containing general information about the disease, while the “Tailored Group” (T) received tailored informative brochures about hypertension (see the following section for details about the brochures’ content). “Non-Material Group” (NM) did not receive any printed information material and was in the condition of usual care.

Ten days following the questionnaire completion, the first two groups of patients received tailored (experimental group T) or generic (experimental group NT) informative materials at their homes. This procedure was repeated in the first follow-up conducted six months after baseline. During the second follow-up, twelve months after baseline, patients were contacted by telephone and asked to respond to an interview that collected information about physical activity.

The sample size was calculated a priori by resorting to power analysis [22], through G*Power Software Version 3.1.9.7 [23], a widely used statistical program for the social, behavioural, and biomedical sciences. The sample size required was calculated to perform a repeated measures ANOVA with the following parameters: f = 0.25 (medium effect size), α = 0.05, power = 0.95; number of groups = 3; number of measurements = 3. The sample size calculated was 168 individuals.

The study protocol was approved by the Ethics Committee of the University of Milano-Bicocca and of the hospital where the patients were recruited, and it was retrospectively registered at ISRCTN registry (Trial ID: ISRCTN13415993).

### Tailored communication intervention

Patients in groups T and NT received an informative brochure regarding hypertension at baseline and the six-month follow-up. Both brochures, tailored vs. generic, reported information about six central thematic nuclei: (i) symptoms and risk factors of hypertension; (ii) the possible changes in daily life following the diagnosis of hypertension; (iii) the risks and complications deriving from the pathology; (iv) optimal management of hypertension; (v) drug therapy; (vi) lifestyle.

In order to choose the contents of the information brochures, information materials focused on hypertension were collected, such as brochures, leaflets, and pamphlets. The contents of the brochures were written by the authors of this study (P.S., M.D., E.R.C., and A.G.), all experts in research with patients with CVDs. The contents were then reviewed by physicians who operated in the clinical settings where the study occurred. The brochures were identical in structure, number of pages, and graphic format. Only the degree of customization of the information content varied: generic for the NT group and tailored for the T group.

The Health Action Process Approach (HAPA) [10] was the theoretical model that guided the tailored communication intervention design. It was used to identify patients who were in the volitional or motivational phase and to define the contents of the brochures they received. The underlying idea was that individuals go through different phases on their path to behavioral change. Therefore, interventions may be more efficient if tailored to these phases. Specifically, the content was tailored to the following variables: adherence to pharmacological therapy, illness perception, lifestyle, and the HAPA constructs (intention to change lifestyle, outcome expectancies, risk perception, self-efficacy, action and coping planning) and information need, all variables connected with the behavioral improvement.

Further details on all the variables assessed and manipulated to generate the tailored brochures, including examples, are reported elsewhere [19].

### Assessment of physical activity

Physical activity was assessed through the Italian version of the Rapid Assessment of Physical Activity Questionnaire (RAPA-Q) [24]. According to the American Heart Association [25], this tool is one of the most common and valid in investigating physical activity. It evaluates the respondent’s frequency and intensity of physical activity and aerobic exercise through seven questions with dichotomous answers (no/yes). The different levels of intensity of exercise (light, moderate, intense) are described through concrete examples and cartoons (e.g., “Moderate activity is defined as physical exercise during which the heart beats faster than normal, such as brisk walking, aerobics or calm swimming”) to facilitate the compilation. The total score ranges from 1 (i.e., sedentary) to 7 (i.e., regular and vigorous activity), with higher scores indicating a higher amount of physical activity. Scores of 6 or 7 (i.e., at least 30 min of moderate to vigorous aerobic exercise five times a week) indicate the target amount of physical activity for cardiovascular prevention, and it was used as a cut-off to classify patients as adherent or non-adherent to recommendations.

### Data analysis

Analyses were performed using IBM SPSS Statistics, version 26 (SPSS, Chicago, IL, USA) and Mplus software, version 7.0. All statistical tests were two-tailed; a *p* ≤ 0.05 was considered statistically significant.

Descriptive statistics were calculated on the sample’s sociodemographic, clinical, and behavioral characteristics. Mean and standard deviation (SD) were reported for continuous variables, and percentages were reported for categorical variables. The normal distribution of the data was tested by calculating skewness and kurtosis indices; recommended ranges of ± 2 and ± 7 were considered for normality, respectively [26].

Attrition is expected in longitudinal studies [27]. Mann-Whitney U and Chi-square tests were used to identify possible dissimilarities between the sample who concluded the study and the participants lost to follow-up. Moreover, a Little Missing Completely at Random (MCAR) test was run to evaluate if physical activity data from patients who dropped at the follow-ups were missing at random [28].

Preliminarily, a repeated measures ANOVA test was performed to directly compare the three experimental groups’ behavioral changes. A 3 × 3 two-way mixed factorial design was used, with a within-subject factor (the three-time intervals) and a between-subject factor (the three experimental groups).

Subsequently, growth curve models were analyzed to identify the longitudinal change in patients’ behavior at the three time-points. These models, similar to Structural Equation Models (SEM), can trace the temporal trajectory of a variable repeatedly measured over time. The result is a trajectory that summarizes the evolution of a particular behavior. The interpretation of the results is based on two parameters: the intercept and the slope. The first indicates the initial value of the behavior under investigation; the second indicates the change of the curve over time [29]. A significant difference between slope and intercept indicates a change in the analyzed group.

The Full Information Maximum Likelihood (FIML) procedure was applied to estimate the growth models’ parameters. This procedure makes it possible to use all available data, including information from participants with missing data [30, 31]. Therefore, the final number of participants remained equal to 188.

Following McArdle and Nesselroade’s indications [32], the analysis of the growth patterns for each of the three groups of patients was divided into three successive steps. Firstly, a “no growth” model was tested in which the lack of a significant change between the intercept and slope was assumed. Subsequently, a “linear” model was tested, in which a constant change was assumed in the three different follow-ups. The saturations of the parameters on the growth curves were fixed at 0, 1, and 2, indicating the baseline (0), the six-month follow-up (1), and the second follow-up at 12 months (2). Finally, in the third model defined as “free time score,” the change curve was not defined a priori, and the parameter saturations on the growth curves were set only for the first two parameters at 0 and 1. In contrast, the third parameter was freely estimated.

The goodness of fit of the different models was evaluated using the χ², the Comparative Fit Index (CFI) [33], the Tucker-Lewis incremental fit Index (TLI) [34], the standardized root-mean-square residual (SRMR) [35], and the Root Mean Square Error of Approximation (RMSEA) [36]. For the CFI and TLI, values of 0.95 or above indicate a good fit, whereas values of 0.90 and < 0.95 are taken as marginally acceptable fit [37]. Values close to 0.06 for the SRMR and RMSEA indicate a good fit; between 0.06 and 0.08, a moderate fit, and values larger than 0.10 indicate a poor fit.

In testing growth curves, each successive model was nested into previously tested models with lower equivalence constraints. For this reason, if more than one model had adequate adaptation indexes, the selection of the best one was performed based on the delta χ^2^ [38]; in addition, the model with the lowest BIC value [39] was judged as the optimal one.

## Results

### Study sample

The sample consisted of 188 consecutive patients with essential arterial hypertension. They were equally distributed for gender, with 94 women (50%), and they had a mean age of 63 years (range 26–86, SD: 10.89). The majority of patients were retired (73.9%), earned a high school diploma or a lesser degree (96.2%), and were married (76.1%). The complete description of the participants concerning sociodemographic and clinical variables is reported in Table 1.

**Table 1:** Sociodemographic and clinical characteristics of the sample (*n* = 188) at the baseline

Sociodemographic Variables	
Age (yr ± SD; range)	63.26 ± 10.89 (26–86)
Gender	
Male	94 (50%)
Female	94 (50%)
Working status	
Working	49 (26.1%)
Not working	139 (73.9%)
Educational level	
High school or less	176 (96.2%)
University	7 (3.8%)
Marital status	
Single\widowed\divorced	45 (23.9%)
Married	143 (76.1%)
**Presence of risk factors**	
Dyslipidaemia	95 (50.5%)
Smoking	16 (8.5%)
Diabetes	34 (18.1%)
Physical Inactivity (RAPA-Q < 6)	115 (61.2%)

Notes. Means and standard deviations (SD) are reported for age. Frequencies (n) and percentages (%) are reported for gender, working status, educational level, marital status, and each risk factor

As for dropouts, 37 patients (19.7%) were absent at the six-month follow-up, and 62 (33.0%) were absent at the 12-month follow-up. The dropout rate at the 12-month follow-up was similar to that reported in other European studies of patients with cardiovascular diseases [40].

Patients who dropped out of the study did not differ significantly from the participants in their sociodemographic characteristics, namely age (t1: U = 2912.00; z = 0.400; *p* = 0.689; t2: U = 3331.00; z = -1.640; *p* = 0.101), gender (t1: χ^2^(1,188) = 1.649; *p* = 0.199; t2: χ^2^(1,188) = 3.465; *p* = 0.063), working status (t1: χ^2^(1,188) = 0.022; *p* = 0.882; t2: χ^2^(1,188) = 0.088; *p* = 0.766), education (t1: χ^2^(1,188) = 1.782; *p* = 0.182; t2: χ^2^(1,188) = 1.188; *p* = 0.276), and marital status (t1: χ^2^(1,188) = 0.849; *p* = 0.357; t2: χ^2^(1,188) = 0.003; *p* = 0.954).

Patients present at the baseline and those who participated at the final follow-up did not differ significantly in their physical activity (U = 3696.50; z = -0.613; *p* = 0.540). Statistically significant differences were found in physical activity between patients present at the baseline and those who participated at the first follow-up (U = 2173.00; z = -2.145; *p* = 0.032). Patients who participated at the first follow-up were more physically active (mean = 4.40) at the baseline than patients who dropped out (mean = 3.70).

The MCAR test was not significant (χ^2^(3,188) = 5.118; *p* = 0.163). The non-significant effects of the missing data pattern (dropouts vs. completers) suggested that physical activity data were missing at random and that the estimates of effects were unbiased by the presence of dropouts [41].

### Physical activity: repeated-measures ANOVA

A two-way repeated-measures ANOVA test was preliminarily performed with a mixed design on the total number of patients. The group was included as the between factor. The Mauchly sphericity test was significant (W = 0.506, *p* < 0.001), suggesting a violation of the sphericity assumption. Consequently, the Greenhouse-Geisser correction was used. The results revealed no significant effects of time (*p* = 0.270) or time x group (*p* = 0.247). Table 2 reports a full description of the mean physical activity scores and their variations over time.

**Table 2:** Descriptive statistics of physical activity scores and their mean variations over time

	Group	T0	T1	T2	Δ (T1 - T0)	Δ (T2 - T0)
Mean (% adherence)	T	4.05 (38.7%)	4.31 (41.5%)	4.52 (45.2%)	0.38	0.48
	NT	4.70 (49.2%)	5.10 (57.8%)	4.93 (55.0%)	0.36	0.23
	NM	4.23 (29.2%)	4.11 (32.1%)	4.07 (38.6%)	-0.25	-0.16
SE	T	0.30	0.28	0.29	0.24	0.25
	NT	0.31	0.28	0.30	0.21	0.26
	NM	0.29	0.27	0.28	0.25	0.28

Notes. T = Tailored group; NT = non-tailored group; NM = non-material group; T0 = Baseline; T1 = 6-month follow-up; T2 = 12-month follow-up. Percentage of adherence represents the percentage of patients who reached the target amount of physical activity for cardiovascular prevention (scores of 6 or 7)

At the baseline, 38.8% of patients achieved the target physical activity. This percentage rose to 43.0% at 6-month follow-up and 46.0% at 12-month follow-up. The mean physical activity score was 4.26, 4.54, and 4.49, respectively, on a scale from 1 to 7. The descriptive statistics divided in function of the experimental group (Table 2) suggested differences in the longitudinal trend of the mean physical activity depending on the experimental group. In particular, the tailored group showed a progressive increase in physical activity over time. The non-tailored group showed increased physical activity at the 6-month follow-up, which was not maintained at the 12-month follow-up. The non-material group showed a slight decrease in the mean physical activity levels over time, although the percentage of patients reaching the target physical activity increased. However, this observation was not supported by statistical significance.

### Physical activity: growth curve models

**Fig. 2:**
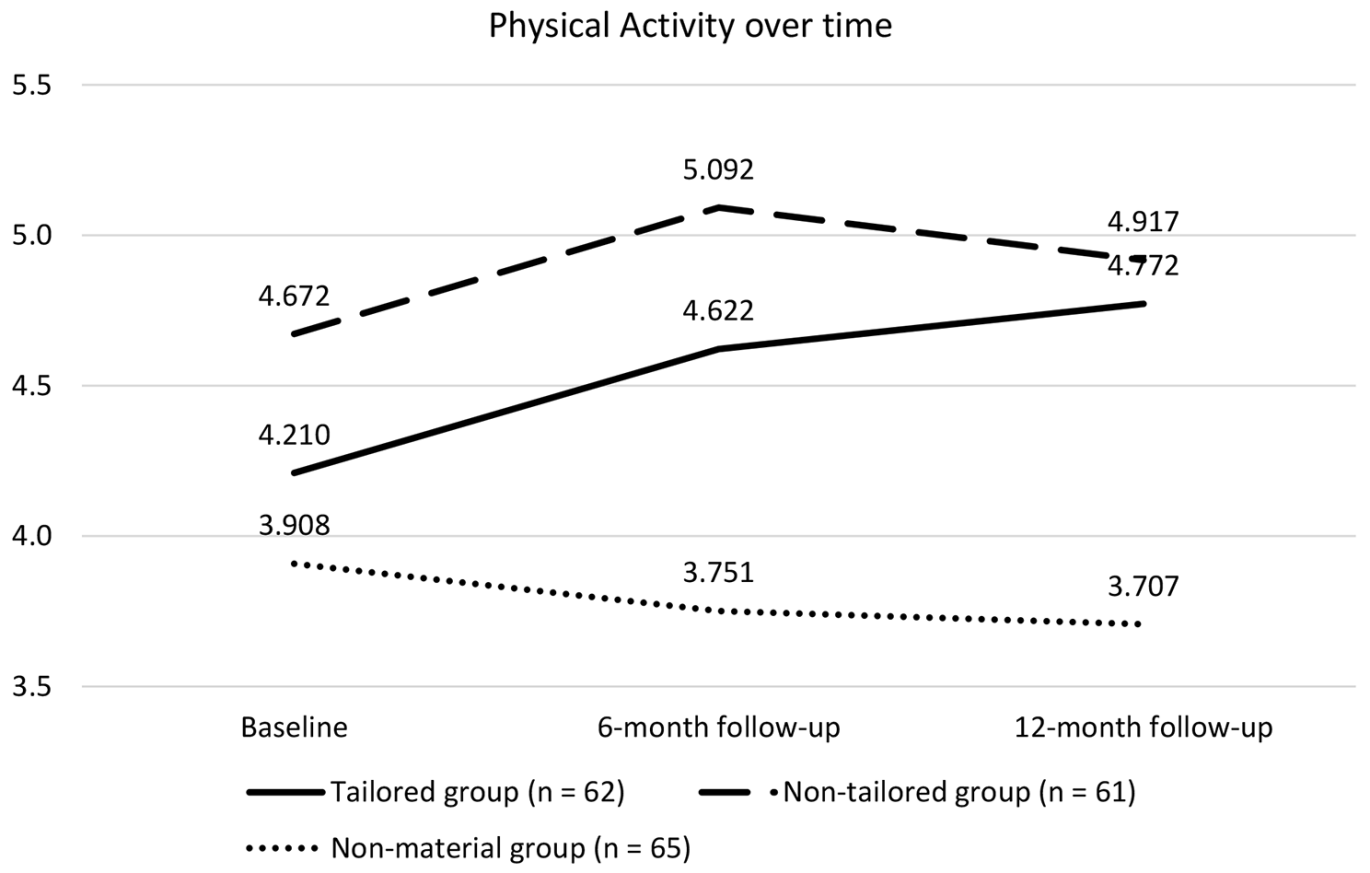
Mean score of physical activity of the three experimental groups for each time-point Notes. Mean scores are different than those showed from the repeated-measures ANOVA model, as a result of the Full Information Maximum Likelihood (FIML) procedure applied for the growth curve model tested

The analysis of the growth curves relating to physical activity began exploring the mean values of the RAPA-Q score in the three-time points. Regarding the tailored group, the mean physical activity score increased from the baseline to the six-month follow-up and slightly increased from the 6- to the 12-month follow-up (Fig. 2). Regarding the non-tailored group, the mean physical activity score increased from the baseline to the six-month follow-up but slightly decreased from the 6- to the 12-month follow-up. Regarding the non-material group, a slight negative swing in physical activity score over time was observed. Figure 2 illustrates these observations.

The results of the three models (i.e., no-growth, linear, and free-time score) are shown in detail in Table 3.

**Table 3:** Fit indexes of the growth curve models for the three experimental groups

Group	Model	χ^2^ (df)	*p*	CFI	TLI	SRMR	RMSEA	BIC
Tailored	No growth	9.317 (4)	0.054	0.910	0.932	0.095	0.146	577.836
	Linear	4.555 (3)	0.208	0.974	0.974	0.120	0.091	574.054
	Free time score	3.770 (2)	0.152	0.970	0.955	0.121	0.119	574.251
Non-Tailored	No growth	9.205 (4)	0.056	0.949	0.961	0.100	0.146	484.469
	Linear	9.104 (3)	0.028	0.940	0.940	0.100	0.183	485.332
	Free time score	2.245 (2)	0.325	0.998	0.996	0.113	0.045	479.439
Non-Material	No growth	19.682 (4)	< 0.001	0.929	0.947	0.362	0.246	479.138
	Linear	17.391 (3)	< 0.001	0.935	0.935	0.359	0.272	477.874
	Free time score	17.207 (2)	< 0.001	0.931	0.897	0.352	0.342	478.716

Regarding the tailored group, the linear model showed the best fit to the data. Indeed, it presented a non-significant χ^2^, the highest CFI and TLI, and an acceptable RMSEA. The Δχ^2^ test showed a significant difference between the linear model and the no growth model (Δχ^2^ = 4.762; Δdf = 1; *p* = 0.029), whereas the free time score model did not differ from the no growth model (Δχ^2^ = 5.547; Δdf = 2; *p* = 0.062). Furthermore, the linear model showed a better fit to the data when compared to the others for the BIC index.

Regarding the non-tailored group, the free time score model showed the best fit to the data. This model was the only one with a non-significant χ^2^; it had the highest CFI and TLI and a good RMSEA. The Δχ^2^ test showed a significant difference between the free time score model and the no growth model (Δχ^2^ = 6.960; Δdf = 2; *p* = 0.031), whereas the linear model did not differ from the no growth model (Δχ^2^ = 0.101; Δdf = 1; *p* = 0.751). Furthermore, the free time score model showed the lowest BIC index.

Regarding the non-material group, the three growth models did not present acceptable values for any of the fit indices, except for the CFI and the TLI, which overall were better in the no-growth model. The Δχ^2^ test showed a non-significant difference between the linear model and the no growth model (Δχ^2^ = 2.291; Δdf = 1; *p* = 0.130), nor between the free time score model and the no growth model (Δχ^2^ = 2.475; Δdf = 2; *p* = 0.290). Therefore, the no-growth model was identified as the most suitable model to represent the data.

## Discussion

This study aimed to preliminarily evaluate the effectiveness of a theory-based, randomized, controlled, tailored health communication intervention to promote physical activity in patients with essential arterial hypertension. Patterns of physical activity change were investigated within and between three parallel groups of patients randomly assigned to test the hypothesis that the participants who received tailored printed informative materials improved physical activity longitudinally more than individuals receiving generic informative materials or no materials.

For this purpose, two different statistical techniques were implemented, which made it possible to better understand the curve of change in behavior over time within each experimental group and to compare the average trend over time of the three experimental groups.

At mean scores observation of the RAPA-Q scale, the two groups that received the informative material, tailored or generic, showed an increase in physical activity at the six-month follow-up, whereas the usual care group showed a slight decrease. At the 12-month follow-up, the tailored group showed a further increase in physical activity, while the non-tailored and usual care groups showed a decrease.

Based on these observations, a two-way repeated-measures ANOVA was preliminarily conducted with a mixed design on the total sample size, and including the group as between factor. Following post-hoc corrections, results evidenced no significant difference in the mean physical activity over time between the three experimental groups.

Although the mean scores observation suggested that receiving tailored information material could encourage increased physical activity over time, the hypothesis here tested still requires further investigations and empirical confirmations. The lack of a significant between-group effect may be ascribed to the amount of missing data that led the statistical model to include a limited sample size (*n* = 126), which was lower than the one estimated through power analysis (*n* = 168) to detect medium-sized effects.

Attempting to overcome this limitation, the growth curve model was performed making it possible to investigate the longitudinal patterns of the three groups using all the available information, including that from participants with missing data.

This statistical approach allowed to observe significant and longitudinal improvements in physical activity in the tailored and non-tailored groups, differently from the non-material one. Furthermore, it made it possible to underline the effect of tailoring by estimating a linear increase of physical activity in the tailored group over 12 months. In contrast, the non-tailored group showed increased physical activity at six months, which was not confirmed in the 12-month follow-up.

These results support the initial hypothesis that the more tailored the information is provided, the probability that it positively affects behavior increases. The effect of tailoring might be explained by the consequent increase in the patients’ motivation to process, understand and use the message. Consistently, prior works focused on physical activity change have found that the relevancy and persuasiveness characterizing tailored messages play an essential role in eliciting more favorable cognitive appraisals that, in turn, have important implications for subsequent individuals’ cognitions, affect, and behavior [9, 16, 42]. Besides, it must be recognized that the intervention was tailored based on HAPA model principles, namely considering those social-cognitive constructs (i.e., intention to change, self-efficacy beliefs, outcome expectancies, risk perception) along with related levels, which allowed to reach and better address participants’ behavior change according to their behavior change phase (i.e., motivational or volitional stage). Prior interventions focusing on physical activity promotion and adopting the same theoretical framework suggested significant improvements in physical activity behavior and related outcomes when specifically targeting the psychological and social-cognitive factors underlying behavior change [43–45]. Following this line, taking into account information needs and preferences when aiming to deliver efficacious health promotion contents is of paramount concern, especially among clinical populations where it was shown that the effective provision of appropriate health information is associated with enhanced patient adherence to medical recommendations, behavioral change, better patients satisfaction with their clinical condition, and increased self-care, which are all crucial aspects for disease prevention [20, 46]. As highlighted in the introduction, individuals with hypertension often fail to change their unhealthy habits [2, 5] despite arterial hypertension is known to represent one of the main risk factors for CVDs [2], and despite all international guidelines agreeing to consider a physically active lifestyle as an essential element to prevent or manage the disease [3]. Therefore, the results of this study offer valuable insights into the definition of effective health promotion content, along with informative evidence on the importance of considering the psychological and social-cognitive factors underlying health behavior change.

Nevertheless, despite these results appear encouraging, the effectiveness of the intervention implemented in the present study should be considered partial and not generalizable, as no significant direct between-group comparisons emerge over time. A possible explanation may lie in the multi-component format of the intervention. It is recognized that the interventions focused simultaneously on multiple factors connected to lifestyle are more complex than interventions focused on a single behavior. Although they were shown to have potential to provide wider and relevant information to the patient, they require a more significant commitment, more time for reading, and a more intense cognitive effort to understand them. This cognitive overload could increase the patients’ difficulties, leading them to ignore some information that is determinant for intervention efficacy. Most of the research on tailored interventions has mostly focused on a single behavior at a time so far. It is still being determined which of the two intervention modalities is more appropriate, as only few researchers have compared them directly. For example, Parekh and co-authors [47] found that multi-component communication interventions aimed at increasing physical activity and improving nutrition drive more healthy choices than presenting the same information separately. Similar results emerged in the research by Hyman and co-authors [48], who designed an intervention to increase physical activity, improve nutrition and quit smoking. In contrast, Vandelanotte and co-authors [49] and Schulz and co-authors [50] found no differences between the two intervention methods in favoring the increase in physical activity. Future studies are needed to investigate this issue further.

Another important consideration is that several studies have highlighted the difficulty of increasing physical activity in older adults [15]. It must be remembered that the mean age of the study sample was relatively high.

One further aspect that probably influenced the partial effectiveness of the intervention protocol is related to the mean behavior of the patients both at baseline and over study phases. The mean scores observed on the physical activity indicated the adoption of an unhealthy behavior (below the cut-off of adequate physical activity). Even when physical activity increased, the mean score obtained at the two follow-ups did not exceed the cut-off, indicating that the level of physical activity in terms of frequency and intensity was still insufficient. In conclusion, the present results may also be determined by the measurement scale implemented, which did not allow for the detection of small changes in the patients’ habits throughout the intervention period. Regarding this point, future trials may for example implement more ecological methods and materials (e.g., ecological momentary assessments – EMAs) that may enable research to shed light on patients’ behavioral patterns directly in their real-life environments.

This study has some limitations. The first, as mentioned previously, is the high percentage of patients who did not complete the study. Despite limiting, it must be acknowledged that a high attrition rate is typical in longitudinal research targeting behavior change [51]. Even if the analysis of the missing cases confirmed the absence of significant differences between those who continued the research and the dropouts, the total number of missing should not be ignored in the generalization of the results. A second limitation is the relatively low number of participants, especially considering that the number of patients recruited at baseline was divided into three experimental conditions. A further limitation relates to the scarce possible control over the actual usage of the intervention, given that the provision of the intervention through the distribution of printed material did not allow us to verify how in-depth the reading had been, and detect participants’ inclinations to impress or please the intervention provider (i.e., Hawthorne effect). In conclusion, a fourth limitation is the absence of an objective indicator of change, which was evaluated only based on patients’ self-report responses to the RAPA questionnaire. It is well-known that self-report questionnaires are subjected to information bias, like recall or social desirability bias. Despite this, they are widely used in medical and psychological research as they correlate with more objective measurement methods and are widely considered suitable to provide informative insights into a phenomenon [52]. Moreover, they present some other advantages like high practicality of use, clinical and research applicability, and good cost-effectiveness.

Despite its limitations, this study has a highly innovative character in its attempt to verify the long-term effectiveness of a tailored health communication intervention protocol conveyed through printed informative materials and, most importantly, which is theory-based. In recent years, research on tailored communication has increasingly moved towards new technologies, verifying the effectiveness of health information transmitted through smartphone applications. The implementation of digital tools may assist future research and interventions to overcome the limitations raised within the present study. Indeed, the use of computer-based tailoring algorithms may benefit future health communication intervention procedures thanks to the development and delivery of more complex and comprehensive contents. As a result, these modalities would contribute to make health promotion programs more accessible, applicable, and more engaging for the patients [53–55].

Two strengths from the present study are noteworthy. Firstly, the theory-based longitudinal design. As mentioned previously, most studies on this topic adopted a cross-sectional design or explored the efficacy of tailored contents up to 6 months after the intervention [16–18]. Moreover, prior implementation of tailored health communication protocols mainly included a selection of variables that were judged as most suitable for the research purposes and, in most cases, were limited to a pool of socio-demographic and clinical characteristics. The present study not only extended the timeframe of the investigation, providing informative evidence over 12 months, but it also adopted, for the development of the content, a theory-driven approach that encompassed participants’ psychological and behavioral profiles. Secondly, to the best of our knowledge, the present study is the first to investigate among patients with hypertension the longitudinal physical activity patterns following a tailored health communication intervention within this specific theorical framework. This not only further provides literature with informative evidence on the applicability of theory-based health communication interventions, but it presumably also represents a crucial contribution for future development and implementation of prevention strategies that aim at halting the progression of hypertension and lowering CVDs risks.

## Conclusions

Developing a tailored communication protocol is lengthy and more expensive than defining generic information campaigns [6]. Despite this, tailored health communication has shown, also in this study, to be able to induce an improvement in behavior. Indeed, the within-group growth curve models confirmed the hypothesis that the patients receiving the tailored printed messages would show significantly wider improvements in their physical activity over 12 months compared to patients receiving non-tailored printed messages or no printed materials. However, the hypothesis tested here still requires further investigations and empirical confirmations because of the need for a significant between-group effect.

Overall, the results of this study demonstrate the importance of considering individual differences in the design and development of educational and information campaigns that aim to spread knowledge and awareness to large segments of the population. Implementing digital tools may assist future research and interventions in overcoming the limitations raised in the present study, thanks to the development and delivery of more complex and comprehensive content. The combination of the theory-based tailored approach described in this study and mobile technology could make health promotion programs more accessible, applicable, and engaging for patients [53–55].

Collectively, this study has important implications in both clinical and research settings. Non-communicable diseases still represent the main challenge for health policies worldwide. Improving well-being and health throughout life is one of the research topics on which to focus to respond to political priorities and societal changes. The most recent guidelines identify lifestyle changes as the cornerstone of prevention, capable of preventing or delaying the onset of diseases [2, 3]. Above all, prevention means controlling the main risk factors (including lifestyle) and reducing environmental, cultural, social, and psychological barriers that hinder knowledge and the conscious adoption of health behaviors. Therefore, it becomes necessary to develop intervention strategies that, by increasing individuals’ knowledge, awareness, and skills, transform their role from passive subjects to active individuals who propose themselves as interlocutors participating in the care and management of their well-being. To support and facilitate this transformation, it becomes essential to understand how better communicate ‘about health’. The analysis of the peculiarities of the target of communication, including its psychological profile, needs, and preferences should represent the initial moment of any intervention aimed at transmitting information aiming at influencing the intentions and behaviors of individuals.

## Data Availability

The datasets used and/or analysed during the current study are available from the corresponding author on reasonable request.
